# Evolution of the sink and source of dissolved inorganic nitrogen with salinity as a tracer during summer in the Pearl River Estuary

**DOI:** 10.1038/srep36638

**Published:** 2016-11-11

**Authors:** Mei-Lin Wu, Yi-Guo Hong, Jian-Ping Yin, Jun-De Dong, You-Shao Wang

**Affiliations:** 1 State Key Laboratory of Tropical Oceanography and Key Laboratory of Marine Bio-resources Sustainable Utilization, South China Sea Institute of Oceanology, Chinese Academy of Sciences, Guangzhou 510301, China; 2 Marine Biology Research Station at Daya Bay, Chinese Academy of Sciences, Shenzhen 518121, China

## Abstract

In this study, we evaluated the sink and source of the surface water along the PRE using a mixing model method with salinity as tracer. The observational data showed that the decreasing of dissolved inorganic nitrogen (DIN) did not closely follow the physical mixing lines of freshwater and modified seawater. In the western part, DIN consumption by phytoplankton and bacteria uptake (ΔDIN)varied from 15.81 μmol L^−1^ to 88.53 μmol L^−1^. On the contrary, in the eastern part, ΔDIN varied from −63.66 μmol L^−1^ to −10.45 μmol L^−1^. DIN source in the eastern part may be mainly caused by organic matter decomposition, while DIN remove is strongly associated with phytoplankton growth and bacteria consumption. These differential behaviors of the estuary with respect to DIN are largely due to varying degrees of hydrodynamics due to different topography in the two areas. Sensitivity analysis indicated reduction strategies of DIN inputs to coastal waters may improve environment quality in the PRE, due to DIN changes in the freshwater end-member having a determined influence on biological activities (R). Our results indicate that the model may be a valuable way to address the sources and sink of DIN in the river-dominated estuaries.

Nitrogen (N) is an essential element for life in the earth. N inputs are have increased rapidly in recent decades, resulting in increased eutrophication of adjacent coasts^1^. N inputs to estuaries on the Atlantic and Gulf Coasts of the United States are now 2 to 20 times greater than during pre-industrialized times^2^,^3^. Riverine dissolved inorganic nitrogen (DIN) input for the Pearl River Estuary (PRE) and the Changjiang is similar, and a little less than Mississippi River^4^. Nitrogen input caused by human activities can accelerate primary production and eutrophication, resulting in many negative responses, such as increased frequency of harmful algal blooms, hypoxic and anoxic bottom waters, loss of emergent plants, and reduced fish stocks in estuarine and coastal ecosystems^5^,^6^. Potential sources of nitrogen in estuarine area include domestic sewage, industrial wastewater, livestock and poultry breeding excretion, aqua-cultural byproduct, surface runoff, and fertilizer residues^7^. Additionally, atmosphere deposit and bio-fixed nitrogen also are important sources for N input in estuarine ecosystems. On the contrary, nitrogen can be lost to the atmosphere as nitrogen gas through the activities of microbes, or sequestered in sediments. Therefore, N experience the complicate oxidation-reduction cycle because of his different valence ranged from −3 to +5, which may exhibit the different processes responsible for the production and release of nitrogen compounds caused by biological activities and anthropogenic influence. However, N production and consumption mainly involving with physical-chemical environment including physical mixing, salinity gradient and biological activities remain unclear in the complicated estuary.

Estuaries receive continuous inputs of nutrients from human activities in the drainage basin. Nutrients behave different processes in clear and turbid waters. In clear waters, increases in nutrient loading cause predictable increases in the biomass of algae^8^. On the contrary, in turbid estuary, phytoplankton production is often light limited year-round, and growth is low despite the high levels of nutrient input^9^. In Chesapeake Bay and Delaware Bay, there was consistent removal of total N from the water column^10^. The Scheldt estuary appeared to be a consistent net addition (before 1995) or removal (after 1995) of DIN along the estuarine axis^11^. The estuarine nitrogen biogeochemical processes vary spatially and temporarily, and highly also depends on system-specific features. There are important scientific and economic reasons to quantify N addition and remove in the estuaries. These processes are involved with the nutrient supply for primary producers, and environmental problems with potentially large economic impacts in terms of lost sources or costs associated with nutrient removal^10^.

Rapid removal of dissolved inorganic nutrients (especially P and Si) often occurs in the very low salinity (0~5) region of the estuary^12^. Dissolved silicate (DSi) may be mostly buried by sinking of pelagic and benthic diatoms^13^. Dissolved inorganic phosphate (DIP) is strongly particle active, and removed by sediments and suspended matter^14^,^15^. However, Dissolved inorganic nitrogen (DIN) is less particle active but may be removed by biological activities such as denitrification^16^. That is to say, DIN remove may be different from the nutrients (P and Si), with special mechanism in the specific estuary. An interesting question is raised then, that is, how does DIN production and consumption in the estuarine system. The simple box model is often used to evaluate the sink and source of DIN, regardless of the internal processes. Therefore, it is hard to understand the DIN behaviors spatially and temporally. The linear relationship between nutrient and salinity during freshwater and seawater mixing is an effective way to discern the sink and source of nutrient in the estuary^17^. Total N removal are estimated as ca. 50% in the Chesapeake Bay and Delaware Bay^10^. Phytoplankton uptake, nitrification and denitrification are regarded as a major process responsible for nutrient removal in the estuary^10^,^18^. Additionally, topography, water residence time and environmental factors such as salinity have also determined influence on DIN behaviors in the estuary. Therefore, DIN sink and source strongly depended on specific environmental characteristics in the estuarine systems.

The Pearl River Estuary is a subtropical estuary and the second largest in China based on discharge volume from the Pearl River, through which the Pearl River discharges into the northern South China Sea (NSCS). Rapid urbanization and industrialization has taken place during the past half century in the lower reaches of the Pearl River, called the Pearl River Delta. DIN input into the Pearl River, has increased during the past 30 years because of intense human activities in the delta^19^. Eutrophication and hypoxia in the PRE has seldom happed due to dilution of nutrients by seawater-freshwater mixing and short water residence times^20^,^21^,^22^. The topography of the PRE has mixed features of channels. The four river mouths (Humen, Jiaomen, Hongqimen, and Hengmen) located in the northwest side of the PRE^23^. Shenzhen, Dongguan and Hongkong located in the eastern side of the PRE, while Zhuhai, Zhongshan, Jiangmen in western side of the PRE. Guangzhou lies in the western and northern sides of the PRE (Fig. 1). This difference in the western and eastern sides of the PRE plays a key role in DIN biogeochemical processes.

This paper addresses the sink and source of DIN in the PRE. The mixing diagram (DIN vs. salinity) was used to explore the sink and source of DIN involving with physical mixing and biological activities. This approach can afford the valuable way to understand quantification and mechanistic of DIN behaviors and may eventually allow for the forecasting of estuarine behavior.

## Results

During the sampling period, the PRE was characterized by low salinity (0–20) in summer. Salinity increased southwards across the Lingdingyang, and also increased eastwards Shenzhen and Hongkong (Fig. 2a). This result indicated that the freshwater discharge from four tributaries in western side of the PRE have important influence on surface salinity in these area. Suspended particulate matter is inversely with salinity, with higher in the western and northern parts of the PRE, and lower in the eastern and southern parts of the PRE. SPM at A8 remained high (~40 mg L^−1^), a decrease in SPM was recorded at A15 (Fig. 2b). Spatial distribution of surface temperature showed the similar to the salinity, with higher in the northern part (Fig. 2c). COD displayed the higher value in the eastern part except the station C1 (Fig. 2d).

Figure 3(a) shows the spatial variation of DIN from the north to the south and from the west to the east. It can be seen that the concentration of DIN in northern part of the PRE was generally higher than that in the southern part. This distribution of DIN indicated that it mainly originated from the runoff of Pearl River. DIN was general over 60 μmol L^−1^. In the northern part of the PRE, DIN was over 100 μmol L^−1^. The highest concentration was recorded at observation station A8. The plot of DIN vs. salinity is used to identify physical mixing and other possible biogeochemical processes (Fig. 3b). In order to simplify this question, three end-members (two freshwater end-members, modified seawater) were used to construct conservative mixing lines. These stations (A8 and B1) are considered as freshwater end-members, and the station A15 as modified marine end-member. Salinity (about 25) was considered as marine end-member in the PRE^20^. In August, the freshwater flux into the Lingdingyang by Humen and Jiaomen is roughly estimated as Q_1 _= 150 × 10^8 ^m^3^, while by Hongqimen and Hengmen as Q_2_ = 37 × 10^8 ^m^3^ 24. Thus, the DIN concentration at near zero salinity caused by the Human-Jiaomen and Hongqimen-Hengmen is 196.10 μmol L^−1^ 
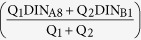
, which is regarded as the new freshwater end-member. Generally speaking, DIN exhibited the clear decreasing trend along the salinity gradient. DIN vs. salinity was concave curve in the western parts, suggesting DIN may be removed by biological activities or dilution by marine water. Nevertheless, DIN vs. salinity was convex curve in the eastern parts, implying DIN may be added by biological activities or external sources.

Over the entire study area, Chl-a concentration decreased along the estuary (Fig. 4a). Especially, Chl-a concentration is higher in northern part (A8-A11, B1 and C1) than the other area (Fig. 4a). Chl-a concentration ranged from 1.41 μg L^−1^ to 21.36 μg L^−1^, and averaged 4.36 μg L^−1^ in summer. The highest concentration (21.36 μg L^−1^) was recorded at A8. Excluding the end-member stations (A8 and A15), Chl-a ranged from 1.41 μg L^−1^ to 5.76 μg L^−1^, with the average value of 3.73 μg L^−1^ in the western and northern parts (Fig. 4b). Chl-a decreased significantly with the salinity gradient in these area (r^2^ = 0.45, p = 0.03). However, Chl-a ranged from 1.55 μg L^−1^ to 3.04 μg L^−1^, with the average value of 2.41 μg L^−1^ in the eastern part (Fig. 4b). Chl-a did not show significant decreasing trend with salinity increasing.

Generally speaking, Chl-a concentration correlated significantly with salinity (R = 0.70, p = 0.03). However, Chl-a concentration correlated insignificantly with salinity in western-northern and eastern parts, respectively. Results of one-way ANOVA for salinity and SPM showed significant spatial variations between the western-northern and eastern parts (p < 0.05), but insignificant difference for DIN and Chl-a.

Both R and ΔDIN were estimated by Eqs 2 and 3. Both of them are positive, implying that biological activities consume DIN in the western and northern parts. R ranged from 5.53% to 55.51%, with average value of about 35.62% except stations A11 and B4 (Fig. 5a). ΔDIN varied from 8.34 μmol L^−1^ to 79.87 μmol L^−1^ (Fig. 5b). By contrast, R ranged from −84.81% to −12.71%, with average of about −37.99% in the eastern parts (Fig. 5a). ΔDIN varied from −63.66 μmol L^−1^ to −10.45 μmol L^−1^ (Fig. 5b).

## Discussion

Many estuaries around the word have extensively experienced intensive nutrient loading in the past several decades^25^,^26^, especially in North America, Northern Europe and economic fast–developing nations^3^, and has become a growing concern in causing coastal environmental pollution. DIN is higher in coastal zone by anthropogenic loadings in the past several decades. Nitrate in the freshwater region ranged between 70 and 120 μM in the Chesapeake Bay^27^, and was up to ~160 μM in the Delaware Estuary^28^. High DIN may lead the deterioration of water quality, ecosystem degradation. To understand the DIN biogeochemical processes is helpful to govern the environment for keeping sustainable development in the estuarine ecosystem. Therefore, in this study, emphasis was made on DIN sink and source in the Pearl River Estuary which is one of the major urbanized estuaries of the world.

The surface water with low salinity in the northern and western parts of PRE is generally characterized by higher DIN. Sewage treatment plants and direct N inputs from surrounding coastal regions, especially from the cities of Guangzhou, Dongguan, and Shenzhen are important DIN sources^29^,^30^. Influenced by the influx of large quantities of suspended solids as well as pollutants in the form of agricultural and domestic wastes from Hong Kong and Shenzhen^31^, turbidity remains high in PRE. The suspended particulate matter (up to 40 mg L^−1^) was higher in low salinity. It is disagreement with previous study^32^. They found that phytoplankton growth can be limited by high turbidity in low-salinity waters (up to 30~150 mg L^−1^ at near-zero salinity in the Pearl River Estuary^33^,^34^. However, Chl-a exhibited the higher value at Humen waters (Fig. 4a). These phenomena have happened in other estuaries. The turbidity maximum and chlorophyll maxima co-occur at intermediate freshwater flows in the North San Francisco Bay^35^. The chlorophyll maximum occurs upstream of the turbidity maximum in the tidal freshwater reaches in the western rivers of Chesapeake Bay^36^. In these zones, the high turbidity does not prevent the accumulation of phytoplankton^10^. Under higher turbid condition, Chl-a concentrations in the east part of PRE tend to be higher and more variable during the summer^37^. The region of the Chl-a maximum was between Humen and Zhuhai (A9-A11, B1 and C1). The Chl-a levels were comparable in magnitude with the previous study and other large estuaries in the world. During summer, the chl-a ranged from 5 to 70 mgm^−2^ in the PRE^38^. Chl-a biomass was up to 126 mg m^−2^ in the euphotic zone of the Chesapeake Bay^39^. The high Chl-a data display significant biological activity as well as biological consumption in PRE. Spatial distribution of observed DIN decreasing with salinity increasing along the estuary suggest that freshwater-seawater mixing occurs in the PRE (Fig. 3a). Previous studies showed that spatail distribution of nitrate in the PRE was controlled by freshwater-seawater mixing. Mixing of the three end-members could be also suggested by a straight line in a plot of DIN vs salinity (Fig. 3b). Nitrate showed the conservative behaviour in the lower reaches of the PRE around the waters south of Lantau Island of Hongkong during July^38^. However, the present study area was up to the Humen throughout the estuary. DIN deviated from the conservative mixing lines, suggesting that N biological consumption or production co-occur in the estuary.

On the basis of the physical mixing diagrams (Fig. 3a), it is noticeable that DIN behaviors were apparently different in the PRE. The Scheldt estuary behaved as a net source of DIN because of its production through mineralization exceeded removal before 1995, and then behaved as a net sink^11^. It is important to identify that DIN change are the net result of remove or opposing processes. Observed DIN in many samples had higher concentrations in the eastern part than would be expected from simple physical mixing, and vice versa in western and southeastern parts. It is likely that DIN would behave in different biogeochemical processes. It is agreement with the previous study^32^. They found the east and west PRE might be associated with different degrees of active internal N cycling.

Based on the results obtained from the Eqs 2 and 3 (Fig. 5), R presented the positive values, suggesting the biological activities consume DIN in the western and northern parts. That is to say, DIN was removed in this area. Chesapeake Bay about one half of the N inputs are lost from the water column, principally via nitrate consumption^10^. Due to complicate topography, the four most distributaries (Humen, Jiaomen, Hongqimen, and Hengmen) in the northern and western parts discharge about half of the Pearl River discharge directly into the PRE^20^,^40^. The southwestward transport of freshwater year-round may reduce the salinity, resulting lower in western and northern parts than the eastern parts (Fig. 2a). The relatively shorter residence time because of high water flow is likely to be one of the limiting factors that result in low phytoplankton biomass in spite of extremely high nutrient concentrations^41^. The Chl-a level remains about 3 μg L^−1^ except station A8, thus DIN may be also removed out of the system by sedimentation of phytoplankton or phytoplankton debris. The chlorophyll was higher in the western parts with higher temperatures, likely indicating that phytoplankton uptake is an important mechanism of DIN removal from the water column (Fig. 2b,c). This phenomenon was also found in Chesapeake Bay^10^. However, relationship between Chl-a and DIN (or ΔDIN) is insignificant difference in the western part (p > 0.05). This result appears to be plausible. Factually, phytoplankton were mainly diatoms that were dominated by *Skeletonema costatum*, *Pseudonitzschia*, *Chaeotoceros* and *Rhizosolenia* in the estuary^42^. Phytoplankton cells in the surface layer die and sink quickly, and eventually sink into the bottom water, and then the diatoms may decompose more slowly due to their rigid silicified cell wall^43^. The vertical mixing is also very limited in the western and northern parts where showed lower salinity and high freshwater discharge (Fig. 2a). Nutrients may effectively bury in the sediment. Meanwhile, the net nitrogen removal efficiency also depends crucially on the nitrogen regeneration from organic matter. Particulate organic matter has a much longer residence time in the estuary than do dissolved substances^44^, as it is moved downstream and upstream associated with the freshwater flow and tidal pumping^11^. Due to high water flow in the western part, particulate organic matter has a much shorter residence time in the western part than in the eastern part. The net effective rate of inorganic nitrogen regeneration from organic matter in the western part may be lower than in the eastern part.

On the other hand, DIN remove by anammox may be not neglected. Under low oxygen, nitrite and ammonium are converted directly into dinitrogen gas by anammox bacteria. However, observed dissolved oxygen in surface water samples in the PRE were 6 mg L^−1^ (data not shown). Anammox bacteria appeared in the water column which did not reach hypoxia in the PRE, and anammox bacteria mainly originated from land soil and wastewater in upstream sites^45^. Denitrification was regarded as a process competing with algal uptake for removal of nitrate from the water column in Chesapeake Bay^10^. As a consequence, Denitrification may play an important role in DIN remove in the PRE. It needs further evidence to support this hypothesis in the further study.

On the contrary, R presented the negative values, indicating DIN is not removed but produced in the eastern part of the PRE (Fig. 5). That is to say, DIN was produced in this area. DIN may be introduced into the ecosystem by different ways such as effluent discharges, atmospheric inputs, recycling from sediments, the decay and breakdown of plant and animal material, from nitrogen fixation and internal source^46^. In estuaries, nitrogen fixation can contribute, 1% of the riverine N input^47^. It is not favorable for N_2_ fixation because of abundant N conditions and high DIN:P ratio (>16:1) in the PRE (data not shown). Atmospheric inputs have been demonstrated to be an important source of nutrients for other regions, including the east coast of the USA^48^. There is not distinguished difference between the eastern and eastern parts of the PRE due to small width. Unfortunately, we have not the related data about nitrogen fixation, atmospheric deposit and effluent discharges, which should further be investigated in the future study.

Unlike the western part, there are not big rivers in the eastern part. Due to the difference between the freshwater flow both sides of the PRE, the residence time of waters in the western part (~0–10 days) is shorter than the eastern parts (10–20 days), particularly during the wet season when river discharge is high^33^,^49^,^50^,^51^. In addition, the east PRE is characterized by the lower flow conditions and stronger tidal dynamics^23^,^49^. Meanwhile, COD presented the higher value in the eastern part (Fig. 2d). It is also favorable for DIN regeneration from organic matter. It would also be tested in future studies.

Additionally, it is also an important aspect of DIN changes of the freshwater and seawater end-members. Next, we elaborate the sensitivity of calculated net effect of biological activities by respective changes of freshwater and seawater end-members in the model. Firstly, DIN concentration of freshwater end-member was changed. Figure 6 showed that the changes of DIN of freshwater end-member in model parameters (±20%) have different effects on different sampling stations. As expected, the higher DIN level was, the more R had (Fig. 6a). Actually, the corresponding changes of R may greatly be large when R was closed to zero (Fig. 6b). The deviation of R even exceeded 100% (station A8). Nevertheless, the DIN concentration of modified seawater end-member was changed; R in higher salinity area had greatly changed more than in the freshwater area (Fig. 6c). The deviation of R totally was less than 60% expect modified seawater end-member (station A15) (Fig. 6d). This result indicated that the freshwater end-member may have a determined influence on this model. Therefore, it also implies that specific strategies for environmental quality of the Pearl River Estuary are needed to effectively reduce nutrient inputs to coastal waters.

Generally speaking, estuarine processes such as DIN source and sink in western and eastern parts can be quite different due to differences in both drainage basin chemistry and anthropogenic influence. The western and northern parts of PRE is less saline and has higher water velocities than the eastern parts, because of the four most distributaries on the northwest bank (Humen, Jiaomen, Hongqimen, and Hengmen) and weaker tidal dynamics^40^,^50^,^52^. All these factors result in different degrees of DIN behavior.

## Conclusion

The simple model involved with DIN vs. salinity is useful way in understanding the sink and source of DIN across the estuarine salinity gradient. Based on this model, the sink and source of DIN in the PRE provided the abundant information about DIN consumption and production during the physical mixing of freshwater and modified seawater. High DIN in the PRE indicated that anthropogenic loading is an important source to the estuary. However, the west-east asymmetric distribution of salinity and DIN may be associated with the respective topography, freshwater discharge and wastewater discharge. The net consumption of DIN (R) in the western part was positive, while negative in the eastern part. It indicated that DIN consumption is mainly consumed by biological activities in the western part, while DIN addition in the eastern part may be mainly caused by organic matter decomposition. These differential behaviors of DIN were related with varying degrees of hydrodynamics and biological activities in the two areas, underlying the sink and source of DIN induced by asymmetric topography. To the western part, the two biological activities remove the DIN including denitrification (organic nitrogen (ON) transferring dinitrogen (N_2_)) and DIN consuming by phytoplankton and sinking-burying in sediments because of high freshwater discharge. To the eastern part, DIN production is mainly associated with organic matter decomposition because of longer residence time and weaker tide dynamics comparing to the western part. Sensitivity analysis indicated freshwater end-member may have a greater influence on deviation of R than in seawater, implying reduction strategies of DIN inputs to coastal waters may be considered to be an effective way to improve environment quality in the PRE. Our study therefore addresses behavior of DIN in the PRE.

## Materials and Methods

### Study area

**Figure 1:**
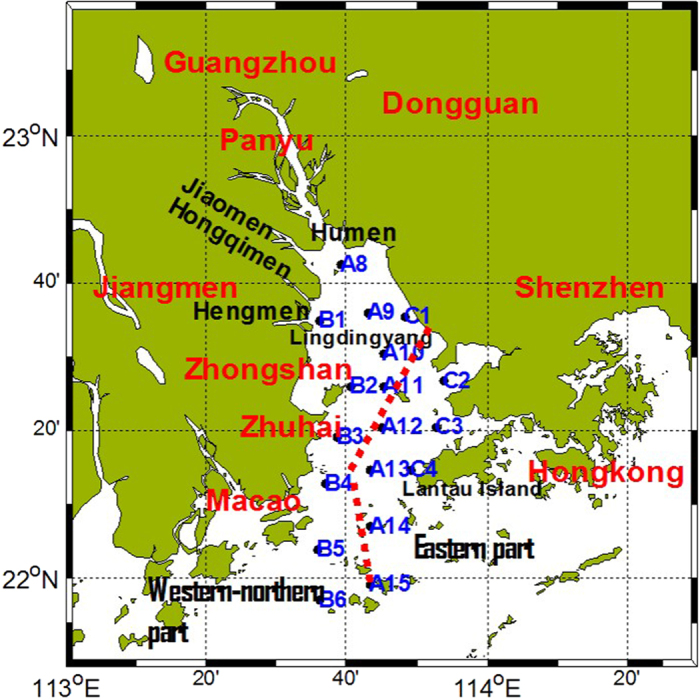
The sampling stations in the PRE. The PRE is divided into the western-northern and eastern parts by the red dotted line. The map in this figure is plotted using MATLAB R2010b ( http://www.mathworks.com/) with M_Map (a mapping package, http://www.eos.ubc.ca/~rich/map.html).

**Figure 2:**
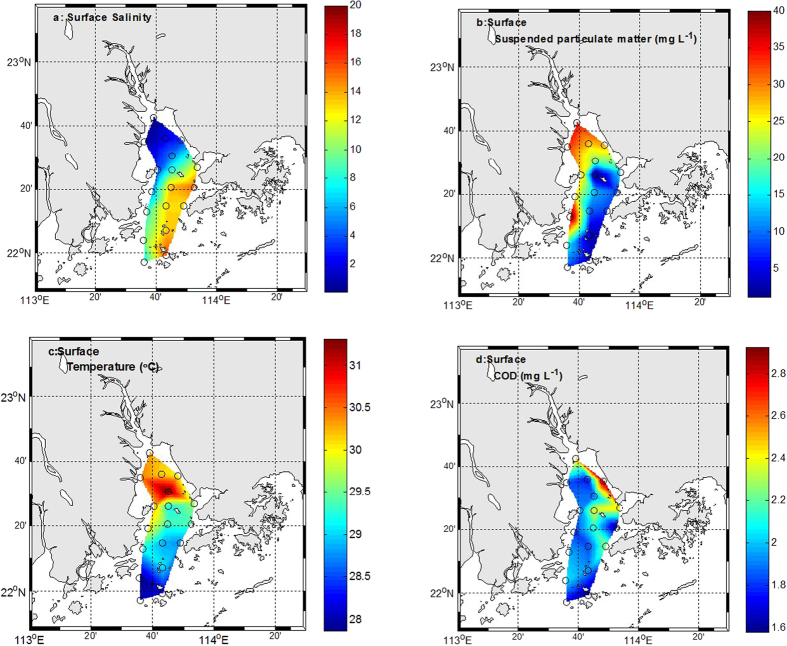
The horizontal distribution of salinity (**a**), SPM (**b**), temperature (**c**) and chemical oxygen demand (COD) (**d**). The maps in this figures are plotted using MATLAB R2010b ( http://www.mathworks.com/) with M_Map (a mapping package, http://www.eos.ubc.ca/~rich/map.html).

**Figure 3:**
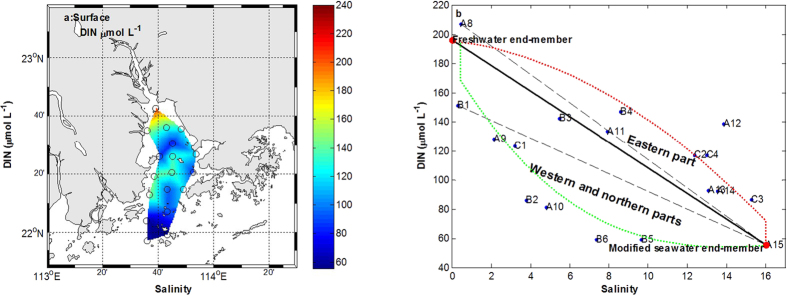
The horizontal distribution of DIN (**a**) and changes in DIN with salinity (**b**). The black dashed line shows the two end-members physical mixing lines for A8-A15 and B1-A15, respectively. The black solid line shows the physical mixing lines for three end-members (A8-B1-A15). The red and green dotted line showed departures from conservative mixing by fitting a high-order polynomial to each of the observed distributions in western and northern parts and eastern parts, respectively. The map in this figure is plotted using MATLAB R2010b ( http://www.mathworks.com/) with M_Map (a mapping package, http://www.eos.ubc.ca/~rich/map.html).

**Figure 4:**
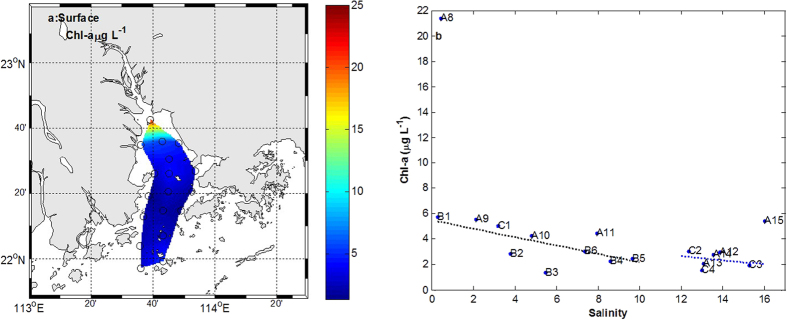
The horizontal distribution of Chl-a (**a**) and changes in Chl-a with salinity (**b**). The blue dotted line shows trend of Chl-a in the eastern part. The black dotted line shows trend of Chl-a in the western and northern parts (r^2^ = 0.45, p = 0.03). The map in this figure is plotted using MATLAB R2010b ( http://www.mathworks.com/) with M_Map (a mapping package, http://www.eos.ubc.ca/~rich/map.html).

**Figure 5:**
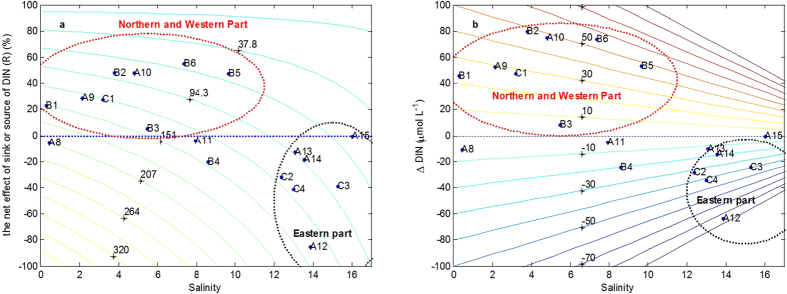
Based on the Eqs 2 and 3, (**a**) simulation of salinity, DIN and biological activities (R) in the PRE. Contour line indicated DIN concentration. (**b**) Simulation of salinity, and biological activities (R) in the PRE. Contour lines indicated the net effect of sink or source of DIN (R).

**Figure 6:**
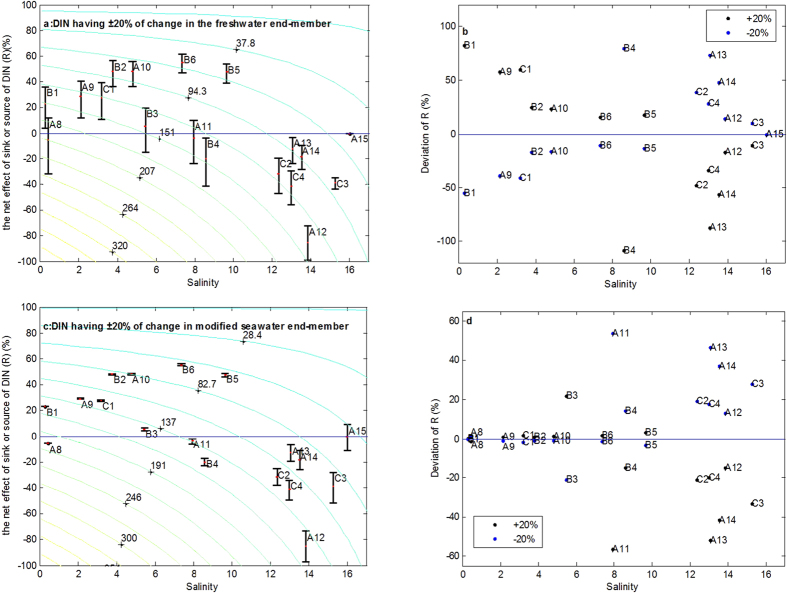
Results of sensitivity analysis. ±20 percentage of changes in DIN levels in freshwater and modified seawater end-members, respectively. DIN levels in freshwater end-member produced the corresponding changes of DIN (**a**) and deviation of R (**b**). DIN levels in modified seawater end-member produced the corresponding changes of DIN (**c**) and deviation of R (**d**). Contour line indicated the DIN concentration when DIN levels remained original value in freshwater end-member (**a**) and modified seawater end-member (**c**), respectively. The red dot indicated DIN levels of sampling stations remained original value in the freshwater (**a**) and modified seawater end-member (**c**), respectively. The black and blue dots indicated the +20 and −20 percentage of DIN changes in the freshwater (**c**) and modified seawater end-member (**d**), respectively.

The Pearl River is China’s third longest river (2200 km), after the Yangtze and Yellow Rivers. The Pearl River has three principal tributaries, namely, the Xijiang River, the Beijiang River and the Dongjiang River^53^. Its drainage basin is located in a sub-tropical climate zone with annual rainfall of 1600–2300 mm^29^. The annual average river discharge is 10,524 m^3^ s^−1^, with 20% occurring during the dry season from October to March and 80% during the wet season from April to September^54^. The Pearl River Estuary is located midway along the northern boundary of the South China Sea. The coast has a NE-SW orientation and the adjacent shelf is 150–250 km wide^19^. The Pearl River stretches for 2214 km and drains an area of 452,000 km^2^ 54. In recent years, rapid economic growth and anthropogenic stress from ambient cities such as Guangzhou, Hong Kong, Macau, Shenzhen, Dongguan, Zhongshan, Jiangmen and Zhuhai have greatly affected the water in the Pearl River Estuary. The environmental quality of the Pearl River Estuary is vital for future sustainable development in the region. In order to find a way to solve the environmental problems of nutrient enrichment and eutrophication, and develop new tools for building ecosystem restoration strategies in Pearl River estuary, it is very necessary to understand the processes of nutrient behaviors. The sampling stations were chosen along the main course axis downstream, covering the whole Lingdingyang (Fig. 1).

### Sampling and analytical method

Water samples were taken at the surface and bottom layers of all stations in August 17–24, 2009. A YSI 6600 V2 Sonde water quality monitoring system (YSI Incorporated, USA) was employed to collect the data for temperature (°C) and salinity (psu) from the surface to bottom layers. Total suspended matter (SPM/ mg L^−1^, detect limit (DL): 2 mg L^−1^) was weighed after drying in a 50 °C oven for 24 h. Seawater samples for analysis of nutrients, chemical oxygen demand (COD/ mg L^−1^, DL: 0.15/mg·L^−1^) and chlorophyll *a* (Chl-a /μg L^−1^, DL: 0.02/μg·L^−1^) were taken using 5-L GO FLO bottles at surface and bottom layers. Water samples from the surface and bottom layers were analyzed for nitrate (NO_3_-N/μmol·L^−1^, DL: 0.05/μmol·L^−1^) and nitrite (NO_2_-N/μmol·L^−1^, DL: 0.02/μmol·L^−1^) with a SKALAR auto-analyzer (Skalar Analytical B.V. SanPlus, Holand). Ammonium concentration (NH_4_-N/μmol·L^−1^, DL: 0.03/μmol·L^−1^) was analyzed with methods of oxidized by hypobromite. Two replicates of 1.0-L samples from the surface and bottom layers were filtered through 0.45 μm GF/F filters and deep frozen immediately at −20 °C. Dissolved inorganic nitrogen (DIN) is sum of nitrite, nitrate and ammonium. At the end of the cruise, all filters were transported to the shore laboratory in liquid nitrogen. Within a week, the chlorophyll was extracted in 10 ml 90% acetone in the dark for 24 h in a refrigerator and its concentration determined with 10-AU Fluorometry (Turner Designs, USA).

### Model buildup

As we well known, salinity is considered as an excellent conservative tracer in the ocean, because it is not created or consumed by chemical or biological processes, when salinity passes through an estuary without undergoing any reaction, except for dilution of freshwater with seawater. In other words, most of substance may behave in a conservative or non-conservative ways in estuarine systems. Therefore, a graph of that substance vs. salinity is used to identify whether behavior of this substance is conservative or non-conservative. If this substance vs. salinity is a straight line which is also known as the theoretical or ideal dilution line, this substance is said to act conservatively. However if the graph deviates from a straight line and becomes curved, implying that the substance reacts by physical, chemical or biological processes, this substance is said to behave non-conservatively^55^. Macronutrients (nitrogen and phosphate) are necessary elements for life growth, so it often is considered as non-conservative substance in marine ecosystem.

In conservative mixing, the concentration of a nutrient can be expressed as a linear function of salinity along a continuum^17^ as followed:


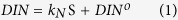


where DIN, k_N_ and DIN^o^, are concentration, slope, and DIN-intercept of nutrients (DIN), in the concentration-S plot, respectively, and S is salinity.

**Figure 7:**
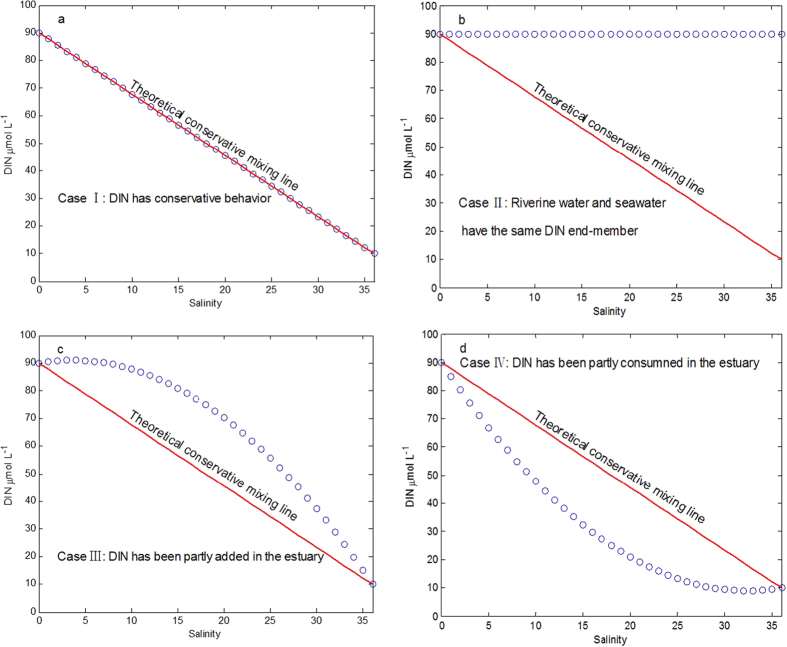
>DIN vs. salinity in four modes of conservative mixing. The red straight line is referred to as the conservative mixing line. (**a**) DIN exhibits conservative behavior; (**b**) DIN has the same value in riverine water and seawater end-members; (**c**) DIN is removed from the system—for example by phytoplankton growth; sedimentation; denitrification; (**d**) DIN is induced into the estuary by different ways, for example from effluent discharges; or from recycling from sediments, or from the decay and breakdown of plant and animal material, or from nitrogen fixation.

The linear correlation between nitrogen concentration and salinity show that the change in DIN concentration is strongly associated with mixing processes across the estuary. On the other hand, indicating that the nonlinear DIN vs. salinity can be caused by biological activities and mixing between riverine water and seawater. Generally speaking, the four hypothetical situations illustrate the trend of DIN vs. salinity in Fig. 7, in order to demonstrate how DIN change by biological activities and mixing along salinity gradient from freshwater to full seawater.

Based on the above-mentioned, salinity is used as a conservative tracer of hydrodynamic mixing between river and seawater, and departures from a linear mixing curve are taken to suggest net uptake (concave upward) or production (convex upward) for a given nutrient^55^. This concept can be useful for investigating the removal of nutrients from estuarine waters by living plants and possible increases in nutrients due to effluent discharges, etc.

To address changes in DIN concentration due to biological activities and physical transport during mixing, amount of loss or gain (sink or source) must be quantified. Because the expected nutrient concentration during conservative mixing can be quantified, the net effect of sink or source of DIN (R) can be expressed as a percentage or fraction of the difference between the observed value and the predicted values of the fitted curves from those of the mixing line. For example, the DIN by physical mixing equals 100 μmol L^−1^, but the observed value is only 70 μmol L^−1^, thus the net consumption of DIN (R) is 30% and the loss of DIN (ΔDIN is 30 μmol L^−1^). Based on Eq. 1, when biological consumption and remineralization are considered, ΔDIN can be expressed as a function of salinity:


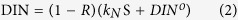



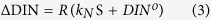


where *R* in Eq. 2 is the net percentage change in DIN, with respect to the conservative mixing value due to the sink or source of DIN (ΔDIN) in Eq. 3. Positive and negative *R* refers to net remove and net addition of DIN.

**Figure 8:**
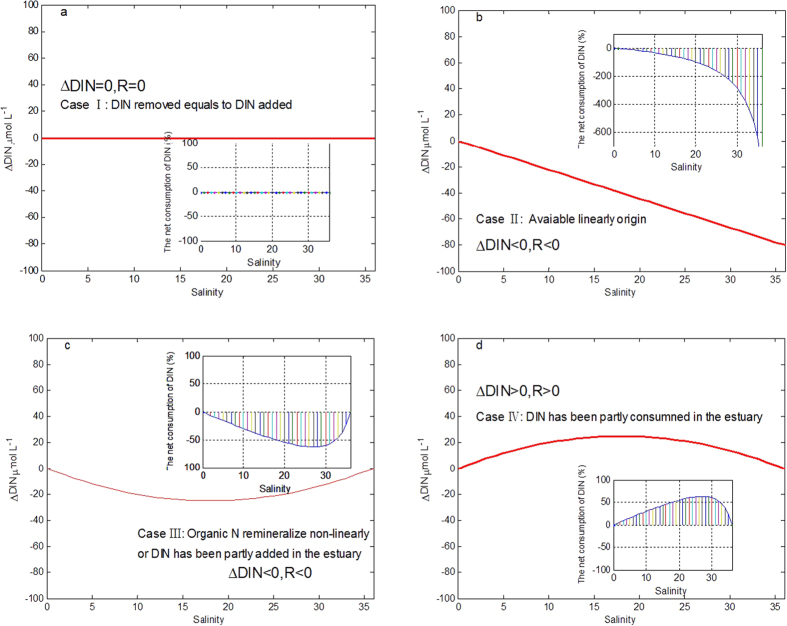
ΔDIN and the net consumption of DIN (%) vs. salinity in four modes of conservative mixing. The red straight line and color lines is referred to as the conservative mixing line and ΔDIN, respectively. (**a**) ΔDIN equals to zero, and the net consumption of DIN is also zero; (**b**) negative ΔDIN increases linearly (R < 0), indicating available linearly origin; (**c**) ΔDIN is negative (R < 0), indicating that DIN is introduced into the ecosystem; (**d**) ΔDIN is positive (R > 0), indicating that DIN is consumed by biological activities and/or physical sediment. ΔDIN vs. salinity shows in the inset figures.

Equations 2 and 3 provide a useful qualitative description of the effect of biological activities and external source such as DIN removed or addition (Fig. 8). Change in DIN due to biological uptake can be described simply as follows. If organisms consume DIN, ΔDIN is positive, namely R more than zero. On the other hand, change in DIN due to remineralization or external input can be described simply as follows. If organic N remineralize or external input, ΔDIN is negative, namely R less than zero. In addition, if the change in the DIN due to the biological consumption and external input during mixing is equal, ΔDIN is will remain constant (zero) and R equals to zero. In order to evaluate sensitivity of net effect of biological activities by respective changes of freshwater and seawater end-members in the model, the changes of DIN of freshwater end-member and seawater end-members in model parameters varied from −20% to 20%, respectively.

**Figure 9:**
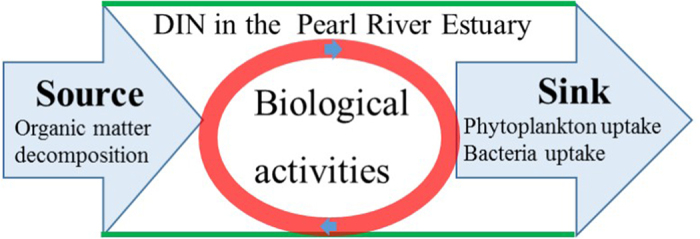
Schematic diagram for source and sink of DIN in the PRE. In the real world, DIN production (source) and consumption (sink) co-occur in the nitrogen cycle. In this study, we just focus on the net effect of source and sink covering above-mentioned three biological activities in the PRE.

Based on above-mentioned theory, the Pearl River Estuary and are taken as the example to explore the sink and source of DIN. In this study, there are one dominant sources of DIN induced by biological activities: organic matter decomposition, and two sinks of DIN: phytoplankton uptake and bacteria assimilation (Fig. 9). To evaluate the source and sink of DIN will be a helpful way to manage the environment development of the PRE.

## Additional Information

**How to cite this article**: Wu, M.-L. *et al*. Evolution of the sink and source of dissolved inorganic nitrogen with salinity as a tracer during summer in the Pearl River Estuary. *Sci. Rep.* **6**, 36638; doi: 10.1038/srep36638 (2016).

**Publisher’s note:** Springer Nature remains neutral with regard to jurisdictional claims in published maps and institutional affiliations.
